# Analysis of epicardial adipose tissue in relation to arterial hypertension using radiomics in photon-counting CT

**DOI:** 10.3389/fcvm.2026.1865937

**Published:** 2026-07-15

**Authors:** Felix Waßmer, Jannik Barz, Dominik Nörenberg, Stefan O. Schoenberg, Alexander Hertel, Isabelle Ayx

**Affiliations:** Department of Radiology and Nuclear Medicine, University Medical Center Mannheim, Heidelberg University, Mannheim, Germany

**Keywords:** cardiac imaging, hypertension, photon-counting CT, radiomics, texture analysis

## Abstract

Cardiac CT is increasingly integrated into clinical routine. However, looking beyond the diagnostic aspect of identifying hemodynamically significant stenosis, individual risk stratification through interpretation of textural alterations of cardiac structures influenced by clinical risk factors, such as arterial hypertension, becomes increasingly relevant after the implementation of photon-counting CT. In this retrospective, single-center, IRB-approved study, 114 patients without coronary artery disease underwent PCCT. Epicardial adipose tissue (EAT) was manually segmented, and 1,015 radiomic features were extracted using PyRadiomics. Patients were divided into two groups defined by the presence or absence of arterial hypertension. Feature selection combined univariate analysis, recursive feature elimination, and random forest importance. Three machine-learning models were evaluated using a leakage-free protocol with repeated stratified cross-validation and a separately held-out test set. Logistic regression achieved the best but only moderate performance (cross-validated AUC 0.65; held-out test AUC 0.59). The most robust quantitative finding was a lower mean EAT attenuation in the hypertension group. These exploratory findings should be regarded as hypothesis-generating and require validation in larger cohorts.

## 1 Introduction

Epicardial adipose tissue (EAT) is a metabolically active fat depot located between the myocardium and the visceral layer of the pericardium. In recent years, EAT has gathered significant attention due to its established associations with coronary artery disease, cardiovascular risk factors, and adverse cardiac events (1, 2). Accurate assessment of EAT volume and distribution has therefore become a topic of growing interest in cardiovascular imaging and research (3, 4). Cardiac computed tomography (CT) represents the modality of choice for EAT assessment, offering high spatial resolution and enabling precise volumetric quantification. The development of photon-counting computed tomography (PCCT) further enhances these capabilities through improved contrast resolution and reduced noise (5). Beyond visual grading of EAT volume and texture, automated segmentation combined with radiomics analysis provides an objective framework for feature extraction. Integrating these—quantitative biomarkers—into PCCT diagnostics may refine individualized cardiovascular risk stratification (6).

Radiomics analysis has become increasingly important in clinical research, driven by the growing interest in transforming subjective and qualitative assessments of medical images into objective and quantifiable data (6). Overall, it is an emerging field in medical imaging that enables the extraction of large numbers of quantitative features from standard radiological images, transforming visual data into high-dimensional, mineable information. In cardiac imaging, radiomics has the potential to characterize tissue heterogenity, morphology, and microstructural alterations beyond what is discernible to the human eye, offering a new dimension to the assessment of cardiovascular diseases (7, 8). Particularly, coronary CT angiography (CCTA) is a widely used non-invasive modality for evaluating coronary artery disease, cardiac morphology, and plaque characteristics. Traditionally, interpretation of cardiac CT images has relied on subjective visual assessment and basic geometric quantification, which may overlook subtle but clinically relevant features and is subject to interobserver variability (7). By contrast, radiomics leverages advanced computational techniques to extract and analyze hundreds to thousands of features—including intensity, texture, shape, and spatial patterns—from regions of interest within cardiac CT datasets (9). Recent studies have demonstrated that cardiac CT radiomics can improve the identification of vulnerable coronary plaques, enhance risk stratification, and provide new imaging biomarkers for early disease detection and therapy monitoring (10). For example, radiomics-based models have shown superior performance in distinguishing acute myocardial infarction from stable coronary artery disease, as well as in predicting major adverse cardiovascular events, compared to conventional CT metrics alone (7). Especially the use of PCCT for EAT radiomics could have a significant impact since studies have already shown that higher radiomics features differ from EICT to PCCT in myocardial assessment (11). Also, radiomics data analysis on PCCT shows a high test-retest stability, making it an encouraging tool for further studies (12).

Despite these promising advances, the integration of radiomics into cardiac CT is still in its early stages, with challenges related to feature reproducibility, standardization of imaging protocols, and validation in diverse patient populations (13). Nevertheless, the growing scientific evidence underscores the potential of radiomics to refine cardiac disease phenotyping, support clinical decision-making, and advance precision medicine in cardiovascular care (14).

In order to determine the clinical relevance of radiomics for individual cardiac risk assessment, this retrospective study aims to evaluate PCCT radiomic features of EAT in patients without coronary stenosis or coronary calcifications in relation to arterial hypertension as a cardiac risk factor.

## 2 Material and methods

This single-center retrospective study analyzed 726 patients who underwent cardiac CT on PCCT between August 2022 and November 2024 at the University Medical Center Mannheim following the current European Society of Cardiology (ESC) guidelines (15). Patients were eligible for inclusion if they had no evidence of coronary artery calcification (Agatston score = 0) and no coronary stenosis on cardiac CT.

Out of 726 patients, 131 met the criteria for study inclusion. All examinations were reviewed by a board-certified radiologist with more than 10 years of experience in cardiothoracic imaging. After further excluding 17 patients because of insufficient image quality or metal artifacts, the study population consisted of 114 patients. Patients included had a median age of 53 years, ranging from 18 to 80 years, with 41 men and 73 women in total. The presence or absence of cardiovascular risk factors such as smoking, diabetes mellitus, and arterial hypertension was recorded before the CT scan using a dedicated questionnaire. The study was conducted in accordance with the Declaration of Helsinki and obtained approval from both the institutional review board and the local ethics committee (Ethics committee II, University Medical Center Mannheim, Heidelberg University, ID 2021-659).

**Figure 1:**
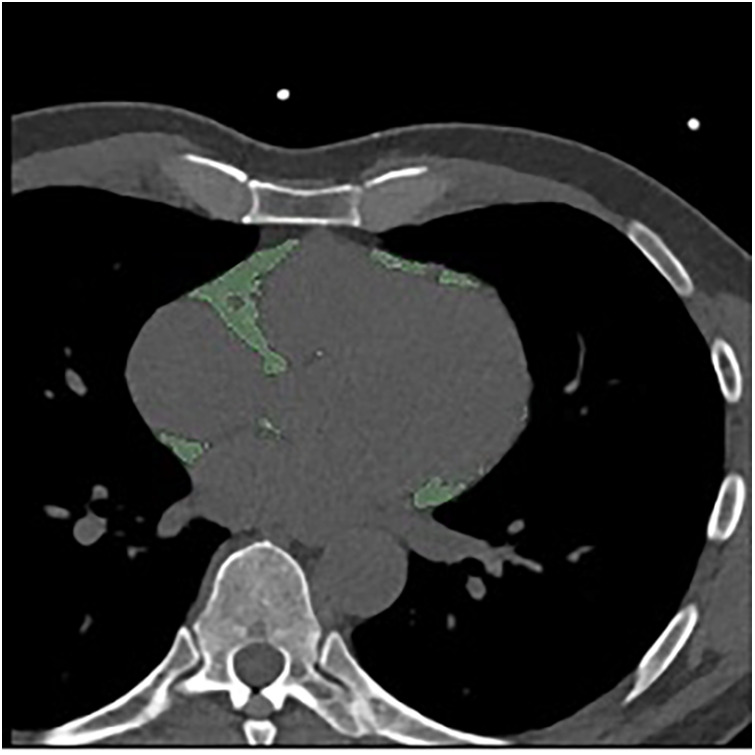
Example CT segmentation of a 69-year-old male patient with epicardial adipose tissue highlighted in green.

All patients included received a non-contrast cardiac CT for CAC evaluation, followed by a contrast-enhanced cardiac CT, both on a first-generation whole-body dual-source PCCT (NAEOTOM Alpha; Siemens Heathineers AG, Forchheim, Germany), using a prospective or retrospective ECG-gated scanning mode (depending on heart rate/heart rate variability). Tube voltage was 120 kV, and automatic dose modulation with CARE keV BQ setting of 64. The effective gantry rotation time was 0.25. Patients received up to 15 mg of intravenous metoprolol and up to 0.8 mg sublingual Nitroglycerin as premedication for reducing heart flow and vasodilatation. Non-contrast-enhanced cardiac CT (axial reconstruction consisting of 3 mm slice thickness, 3 mm increment, and a Qr36 kernel) was performed for evaluation of coronary artery calcifications using a dedicated software (Syngovia, Siemens Healthineers AG, Forchheim, Germany). Contract-enhanced CT (axial reconstructions using 0.6 mm slice thickness, 0.4 mm increment and a Bv40 kernel) was performed using 70–80 mL of iodine contrast (Imeron 350, Bracco Imaging Deutschland GmbH, Konstanz, Germany) followed by a 30–40 mL saline chaser (NaCl 0.9%) with a weight-based flow rate of 4.5–6 mL/s through the antecubital venous access. Coronary CTA was triggered by Bolus tracking in the ascending thoracic aorta with a threshold of 140 Hounsfield units (HU) at 90 kV. Image data was anonymized and converted to Neuroimaging Informatics Technology Initiative (NIFTI) file format for use with a dedicated segmentation tool (3D Slicer, Version 5.2.2, free software license). EAT was manually segmented by a medical student and subsequently reviewed by a board-certified radiologist with 10 years of clinical experience in cardiovascular imaging and 8 years of segmentation expertise. The segmentation encompassed the entire EAT, applying a CT attenuation range of −190 to −30 HU, consistent with established literature (16). An example segmentation is illustrated in Figure 1. The cranial boundary was defined at the midpoint of the pulmonary trunk, while the caudal boundary was set at the interface between the cardiac apex and the diaphragm.

A total of 1015 radiomic features were extracted from the segmentated EAT volumes using PyRadiomics (version 3.1.0, Harvard Medical School, Boston, MA, USA) to capture a comprehensive spectrum of image texture characteristics. Extracted features comprised first-order statistics (*n* = 18), gray-level co-occurrence matrix (GLCM, *n* = 24), gray-level run-length matrix (GLRLM, *n* = 16), gray-level size-zone matrix (GLSZM, *n* = 16), gray-level dependence matrix (GLDM, *n* = 14), and neighborhood gray-tone difference matrix (NGTDM, *n* = 5) features. In addition, multiscale texture information was captured through wavelet-transformed features and Laplacian of Gaussian (LoG)-filtered features with sigma values of 3.0 and 5.0 mm.

To assess the intraobserver reproducibility of the extracted features, EAT was segmented a second time in 10 patients by the same reader several months after the initial segmentation and blinded to it. Radiomic features were extracted from both segmentations using identical preprocessing settings, and their agreement was quantified with the intraclass correlation coefficient using a two-way random-effects, single-measurement, absolute-agreement model [ICC(2,1)]. ICC values were interpreted according to Koo and Li (poor < 0.50; moderate 0.50–0.75; good 0.75–0.90; excellent ≥ 0.90) (17).

The radiomic feature matrix contained no missing values; a median-imputation step was nevertheless included in the pipeline for robustness. To avoid information leakage, all preprocessing and feature-selection steps were encapsulated in a single pipeline that was refit exclusively on the training data within each cross-validation fold. Within every training fold, continuous features were standardized (z-score), and feature selection was performed using the same three-method consensus strategy as described above (univariate *F*-test, top 20; recursive feature elimination with logistic regression, top 15; random-forest importance, top 20; features selected by at least two of the three methods). Three classifiers—logistic regression, random forest, and a support-vector machine with a radial-basis-function kernel—were then trained on the selected features.

Model performance was evaluated in two complementary, leakage-free ways: (i) repeated stratified cross-validation (5 folds × 10 repeats), and (ii) a clearly separated test set, evaluated over 50 repeated stratified 80/20 hold-out splits in which feature selection and model fitting were confined to the training partition and performance was assessed on the untouched test partition. Hyperparameters were tuned by nested cross-validation. Reported metrics comprise the area under the ROC curve (AUC), accuracy, balanced accuracy, sensitivity, and specificity, each summarized as the mean with 95% confidence interval across resampling iterations.

**Figure 2:**
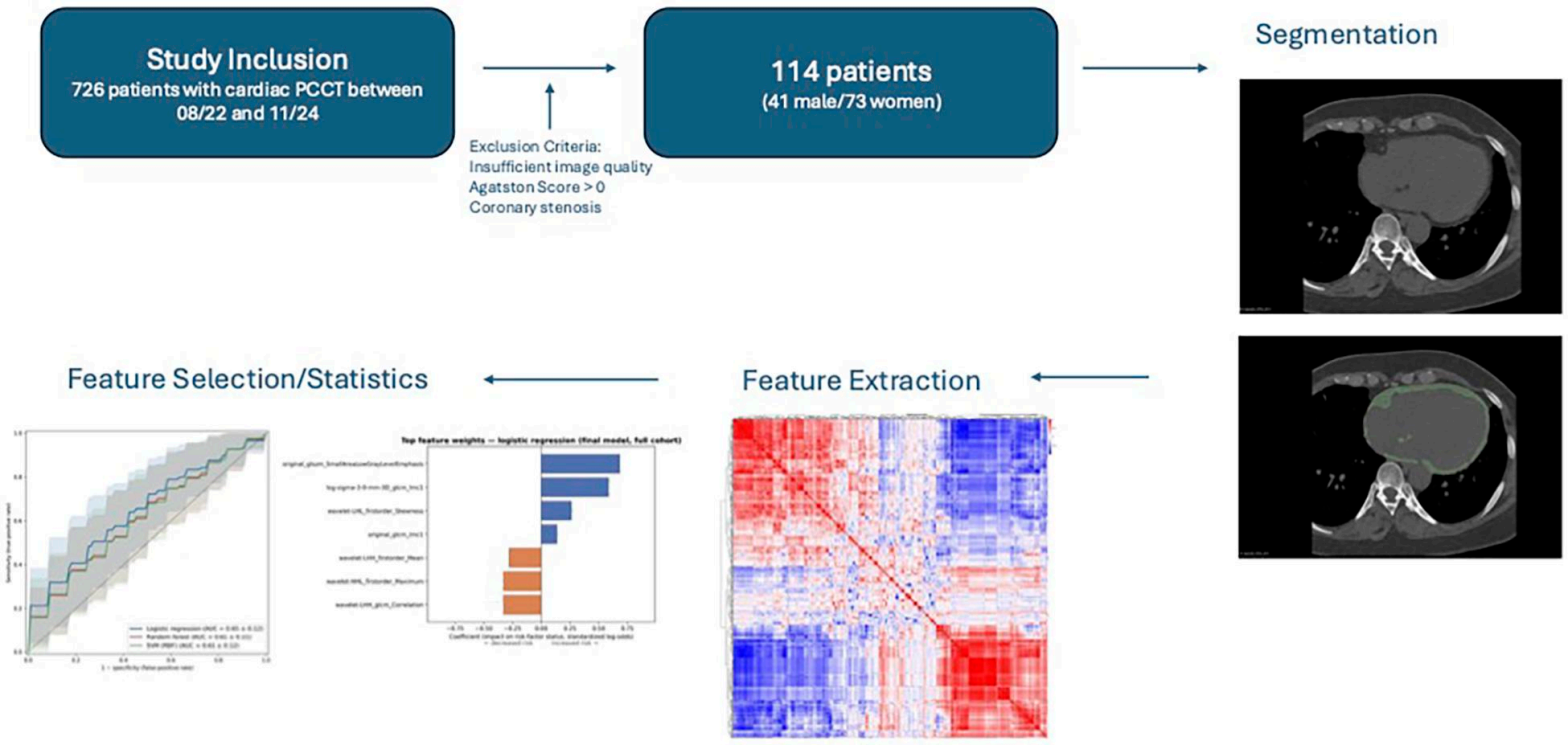
Overview flowchart of patient enrollment, segmentation, and radiomic extraction workflow.

To assess whether the radiomic associations were independent of baseline imbalances, each z-standardized radiomic feature was entered as the dependent variable in a multivariable linear model with hypertension as the predictor, adjusted for age, sex, and diabetes mellitus; *p*-values for the hypertension term were corrected across all features using the Benjamini-Hochberg procedure. Figure 2 outlines the workflow process of this study.

All analyses were performed using Python version 3.11. The following packages were employed: scikit-learn (v1.3.0) for machine learning, pandas (v2.0.3) for data handling, NumPy (v1.24.3) for numerical operations, SciPy (v1.11.1) for statistical testing, matplotlib (v3.7.1), and seaborn (v0.12.2) for visualization.

## 3 Results

A total of 114 patients were enrolled in this study, including 41 men and 73 women, with a median age of 53 years, ranging from 18 to 80 years of age. All patients had no coronary stenosis with a calculated Agatston Score of 0. Approximately 90% of the CT scans were performed in prospective high-pitch or adaptive sequence mode, while about only 10% were acquired retrospectively. Available clinical data regarding risk factors are shown in Table 1. *P*-values for group comparisons were calculated using either Fisher's exact test or the Chi-square test, as appropriate. While both patient cohorts had similar rates of dyslipidemia, nicotine abuse, and COPD, the number of patients with diagnosed diabetes mellitus significantly varied between the two groups with 9 patients in the hypertension group and 1 patient in the non-hypertension group (*p* < 0.05). Additionally, baseline comparison showed that sex (*p* = 0.032) and age (*p* = 0.029) differed significantly between both groups.

**Table 1:** Comparison of different cardiovascular risk factors (diabetes, dyslipidaemia, nicotine abuse, and COPD) for patients with and without arterial hypertension.

Risk factor	Hypertension + (*n* = 55)	Hypertension − (*n* = 59)	*p*-value
Diabetes	9 (16.3%)	1 (1.7%)	0.007
Dyslipidemia	14 (25.5%)	18 (30.5%)	0.677
Nicotine Abuse	19 (34.5%)	18 (30.5%)	0.692
COPD	6 (10.9%)	6 (10.2%)	1.000

Distinct differences in tissue density and volume were observed between the two study cohorts. Patients in the hypertension group exhibited a significantly lower mean tissue density compared to the non-hypertension group, with HU values of −80.31 HU (SD 7.50 HU) vs. −77.56 HU (SD 6.18 HU), respectively (*p* = 0.036). Furthermore, the mesh volume was significantly larger in the hypertensive cohort than in the non-hypertensive control group [95.78 cm^3^ (SD 57.49 cm^3^) vs. 77.40 cm^3^ (SD 37.27 cm^3^), *p* = 0.047].

Intraobserver reproducibility of the radiomic features was excellent. The median ICC was 1.00, with 99.1% of features showing excellent agreement (ICC ≥ 0.90) and the remaining 0.9% good agreement (ICC: 0.75–0.90); no feature had an ICC below 0.77. The few less reproducible features were exclusively wavelet-LLL low-gray-level texture descriptors, which are particularly sensitive to minor differences in segmentation boundaries.

The selection of features was by a consensus between 2 of the following 3 selection strategies or solely by the strongest univariate association: First, univariate feature selection was performed using SelectKBest with the *F*-test for classification (f_classif), re-training the 20 features with the strongest individual associations with the outcome shown in Table 2. “wavelet-LHL_firstorder_Skewness” showed the highest F-score with a value of 12.06 and *p* < 0.01, indicating a robust discriminatory capacity shortly followed by “original_glszm_SmallAreaLowGrayLevelEmphasis” with a F-score of 11.75 and *p* < 0.01.

**Table 2:** SelectKBest top 5 features with *F*-score and *p*-value; number of significant parameters per category.

Rank	Feature	*F*-score	*p*-Value
1	wavelet-LHL_firstorder_Skewness	12.06	0.00073
2	Original_glszm_SmallAreaLowGrayLevelEmphasis	11.75	0.00085
3	wavelet-LHH_firstorder_Mean	11.62	0.00091
4	wavelet-LHH-firstorder_RootMeanSquared	10.02	0.00201
5	wavelet-HHL-glcm_Correlation	9.89	0.00225

No feature reached significance after Benjamini-Hochberg correction (minimum adjusted *p* = 0.30).

Second, RFE was employed in conjunction with logistic regression, iteratively removing the least informative features until the top 15 features remained. Third, feature importance was assessed using a random forest classifier (200 trees), with features ranked according to the mean decrease in Gini impurity. Although the overlap between the top features from each method was limited, a consensus set of 48 features was identified. In the univariate radiomic analysis, 148 of 1,015 features were nominally significant (*p* < 0.05), but none remained significant after Benjamini-Hochberg correction (smallest FDR-adjusted *p* = 0.30).

**Figure 3:**
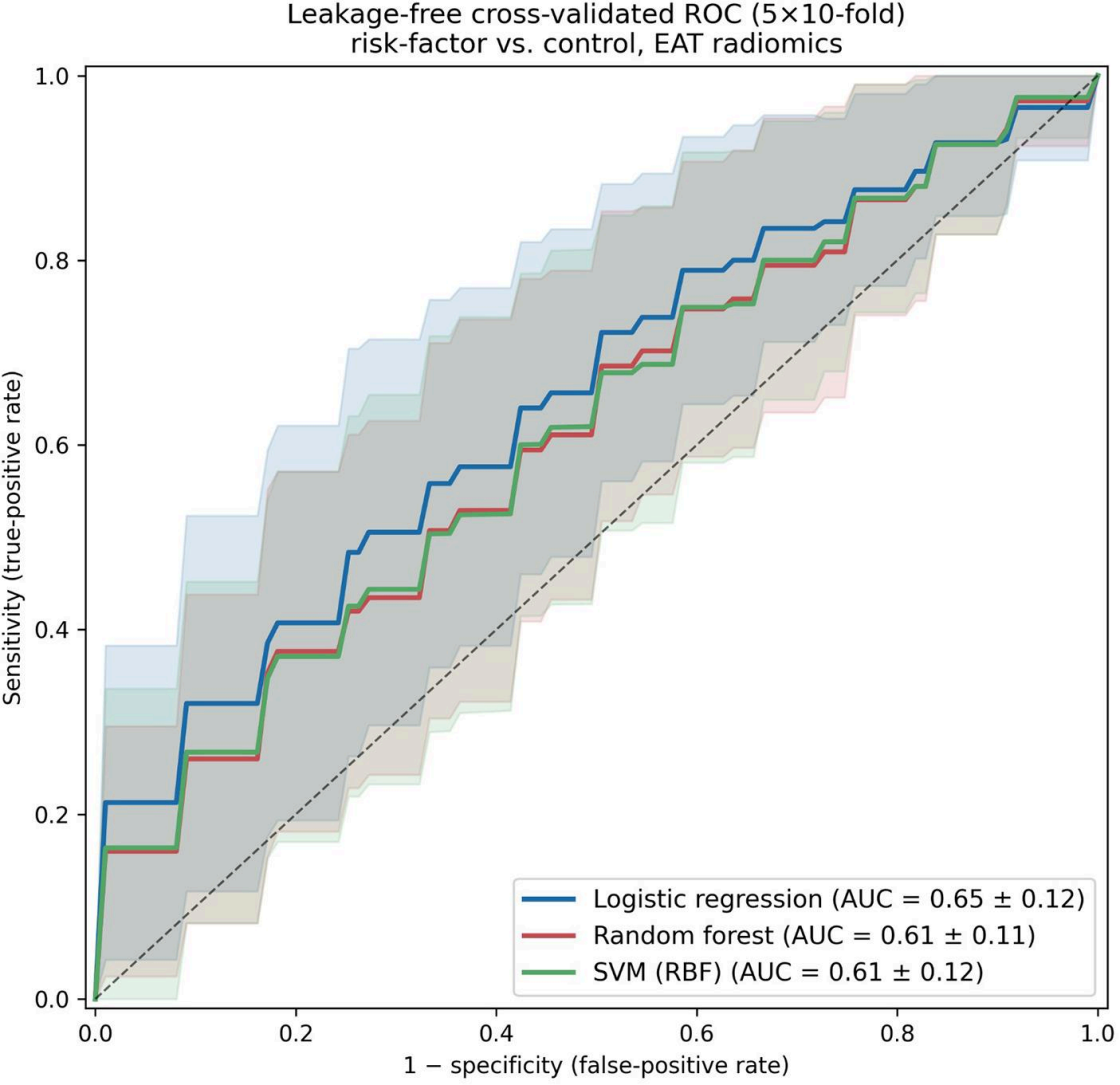
Leakage-free cross-validated receiver-operating-characteristic (ROC) curves for the classification of risk-factor status from EAT radiomic features. Mean ROC across repeated stratified 5 × 10-fold cross-validation for logistic regression, random forest, and SVM; shaded bands denote ± 1 standard deviation across folds. All preprocessing and feature selection were performed within the training folds only. Mean AUC values are given in the legend.

**Figure 4:**
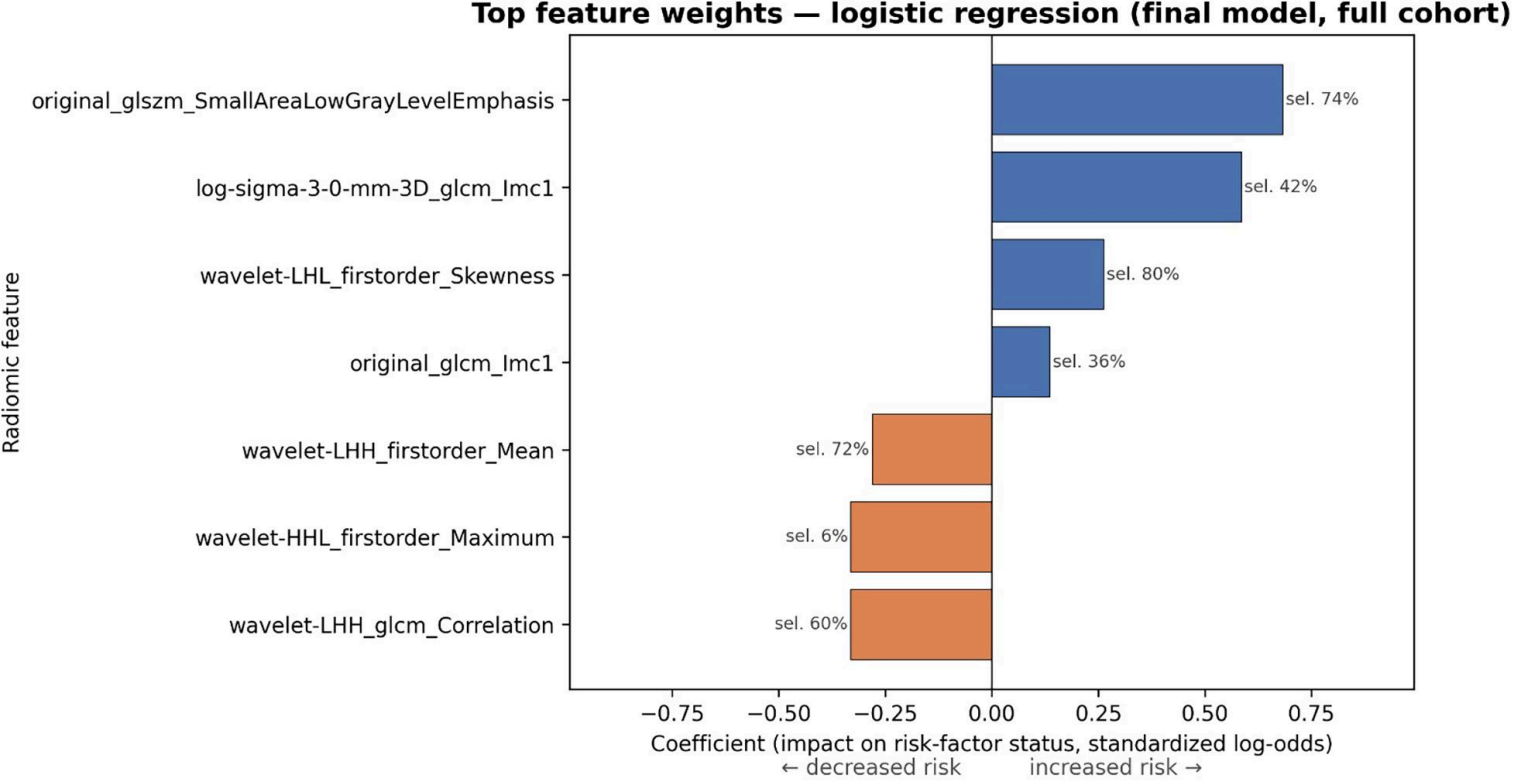
Top features of the logistic regression model with positive and negative coefficient for arterial hypertension.

Under the leakage-free protocol, the three classifiers achieved only moderate discrimination (Table 3). Logistic regression performed best, with a cross-validated AUC of 0.65 (95% CI: 0.61–0.68) and a performance on the clearly separated hold-out test set of AUC 0.59 (95% CI: 0.36–0.75); random forest and SVM yielded comparable values (cross-validated AUC 0.61 each). The corresponding ROC curves are shown in Figure 3. The features with the highest weights in the final (exploratory) logistic-regression model are shown in Figure 4. Small Area Low Gray Level Emphasis carried the largest positive coefficient, suggesting that a higher prevalence of fine-grained, low-intensity texture patterns within the EAT volume was associated with risk-factor status; log-sigma-3 mm GLCM Imc1 and wavelet-LHL firstorder Skewness were further positively weighted features, whereas wavelet-LHH GLCM Correlation and wavelet-LHH firstorder Mean were negatively weighted. These coefficients were derived from a single model fit on the full cohort and are provided for descriptive purposes only. Notably, in the univariate analysis none of the 1,015 radiomic features remained significant after Benjamini-Hochberg correction (smallest FDR-adjusted *p* = 0.29), and the cross-validation selection frequency of individual features varied considerably (Figure 4).

**Table 3:** Statistical performances with area under the curve (AUC) and accuracy values of three machine learning models.

Model	Cross-validated AUC (5 × 10)	Held-out test AUC	Accuracy	Balanced accuracy	Sensitivity	Specificity
Logistic regression	0.65 (0.61–0.68)	0.59 (0.36–0.75)	0.57 (0.43–0.73)	0.56 (0.43–0.73)	0.51 (0.27–0.80)	0.62 (0.42–0.83)
Random forest	0.61 (0.58–0.64)	0.55 (0.31–0.72)	0.52 (0.35–0.65)	0.52 (0.34–0.65)	0.51 (0.18–0.80)	0.54 (0.33–0.81)
SVM (RBF)	0.61 (0.58–0.65)	0.55 (0.30–0.73)	0.53 (0.35–0.65)	0.52 (0.35–0.65)	0.48 (0.27–0.73)	0.57 (0.33–0.81)

In a multivariable analysis with each z-standardized radiomic feature as the dependent variable and hypertension as the predictor, adjusted for age, sex, and diabetes mellitus, 55 of 1,015 features remained nominally associated with hypertension (*p* < 0.05), including the highest-weighted model features (e.g., wavelet-LHL firstorder Skewness, *β* =  + 0.56 SD, *p* = 0.006). After Benjamini-Hochberg correction, however, no feature remained significant (smallest adjusted *p* = 0.75), indicating that the radiomic associations were not robust to multiple-comparison correction even after accounting for baseline imbalances.

## 4 Discussion

This study demonstrates that quantitative characterization of EAT on PCCT reveals measurable differences between patients with and without arterial hypertension, even in the absence of coronary calcifications or stenosis. The most robust finding was a significantly lower mean EAT attenuation in the hypertension group (−80.3 vs. −77.6 HU; *p* = 0.033), accompanied by a numerically higher EAT volume—a pattern fully concordant with conventional CT-derived EAT measurements and consistent with hypertension-associated changes in adipose tissue composition.

Importantly, the exploratory radiomic analysis pointed in the same direction: among more than 1,000 extracted features, texture- and intensity-based parameters, particularly small-area low grey-level emphasis, log-sigma-3-mm GLCM Imc1, and wavelet-derived skewness, carried the highest weights in the logistic-regression model (Figure 4) and reflected the same underlying shift in tissue attenuation and heterogeneity captured by the significant conventional metrics. While none of these individual features retained significance after correction for multiple comparisons, and should therefore be interpreted as descriptive rather than established associations, their directional concordance with the HU and volume findings lends biological plausibility to the overall signal.

After a fully leakage-free analysis, logistic regression achieved the best performance among the tested classifiers, with a cross-validated AUC of 0.65 (95% CI: 0.61–0.68) and a held-out test-set AUC of 0.59 (95% CI: 0.36–0.75), indicating a modest but reproducible discriminative tendency that warrants confirmation in larger cohorts.

A recently published study outlined the significance of considering the EAT radiomics profile in predicting myocardial ischemia. In a large, multicentric cohort of patients with angina pectoris and intermediate-to-high pre-test probability of CAD, the EAT radiomic model outperformed traditional CT-derived metrics—including EAT volume, EAT density, CAD reporting and data system (CAD-RADS), and coronary artery calcium score (CACS)—as well as a combined clinical + CACS model in identifying myocardial ischemia as defined by reduced quantitative myocardial blood flow. The radiomic model demonstrated excellent discrimination, achieving AUC values of 0.840 and 0.838 in the training and external validation sets with a sensitivity of 80% and NPV of 89%, suggesting its potential value as a non-invasive screening tool to rule out ischemia in patients undergoing evaluation for suspected CAD (18). Another study by Lee et al. combined EAT textures with the amount of coronary calcifications. This led to a chance for the successful prediction of hemodynamic relevant CAD. These findings underscore the added value of texture-based analysis of EAT, which may capture subtle inflammatory or compositional changes not reflected by conventional CT parameters (19).

Mundt et al. demonstrated the feasibility of radiomic analysis of EAT to differentiate between patients with and without CAD, when CAD was defined as coronary artery stenosis of equal to or above 50% lumen obstruction. Especially first-order and shape-based radiomic parameters demonstrated significant differences between patients with and without CAD, as confirmed by coronary angiography. The high AUC values for these features—0.753 for Median and 0.775 for Round—suggest that quantitative analysis of EAT texture and morphology may provide meaningful insight into coronary pathology (20).

In a study by Wei et al., four radiomic texture features of EAT were significantly associated with CAC burden, suggesting a potential link between CAC and EAT texture characteristics. Notably, three of these four features demonstrated a dependency on CAC burden, suggesting that radiomic characterization of EAT may reflect biological processes associated with coronary atherosclerosis. Inflammatory remodeling of EAT may lead to reduced tissue density and increased structural heterogeneity, contributing to the altered radiomic profiles observed in patients with CAC (21).

In line with our results, suggesting an influence of clinical cardiovascular risk factors on EAT, Kahmann et al. investigated the influence of hypercholesterolemia on peri-coronary adipose tissue (PCAT). Their study demonstrated that radiomic texture analysis of PCAT can differentiate between patients with and without hypercholesterolemia, suggesting that PCAT radiomics may serve as a novel imaging biomarker for this specific cardiovascular risk factor. Among the extracted features, gldm_HighGrayLevelEmphasis and glrlm_HighGrayLevelRunEmphasis showed higher values in patients with hypercholesterolemia, indicating a greater concentration of high-density voxel values in PCAT. These findings could possibly reflect underlying histopathological changes, such as lipid accumulation, inflammation, or fibrosis, which could influence the attenuation characteristics of PCAT and be captured through texture-based metrics (22).

Another multicenter retrospective study investigated the possibility of radiomic features of PCAT, derived from CCTA, to improve long-term prediction of MACE in patients with ACS. A total of 777 patients from three medical centers were included, with 664 forming an internal cohort used for model development and validation, and 113 serving as an external test set. Multivariable Cox regression models incorporating clinical scores, traditional CCTA findings, PCAT attenuation (PCATa), and PCAT radiomics were constructed. While the addition of PCATa did not enhance predictive performance, integrating PCAT radiomic score—either from the culprit vessel or across all three vessels—significantly improved risk prediction, with consistent performance across training, internal test, and external test sets. The inclusion of PCAT radiomics also led to significant improvements in risk discrimination and reclassification metrics. These findings suggest that PCAT radiomics provide complementary prognostic value beyond conventional clinical and imaging parameters and may enhance long-term risk stratification for MACE in ACS patients (23). In contrast, our study highlights the potential underlying effect of clinical cardiovascular risk factors on EAT, which may lead to a higher risk of CAD and MACE (24), thereby providing a baseline for further research. According to this, Agnese et al. (25) found that patients with a BMI > 25 kg/m^2^ had a significantly higher EAT volume than patients below this BMI. Although having no difference in mean HU, radiomic analysis showed different parameters for this cut-off value, suggesting that a higher body weight affects those textures significantly. With obesity being a main risk factor for arterial hypertension in adults (26), the present results add to a growing body of evidence suggesting that quantitative characterization of cardiac adipose tissue may provide complementary information for cardiovascular risk assessment.

Nevertheless, this study is limited by various factors, including its single-center approach and a relatively small study population, which is a consequence of the relatively novel implementation of PCCT. Furthermore, the hypertension subgroup had significantly (*p* < 0.05) more patients with a diagnosed diabetes mellitus and a significant variation in age and sex of the population, which could not be fully adjusted for given the limited sample size, so that residual confounding cannot be excluded. This may alter the meaningfulness of the results; hence, DM can change radiomic parameters of pericoronary fat tissue (27) and epicardial fat tissue (28), driven by a chronic inflammatory process (29). This inflammation can lead to several biocellular cardiac changes, enhancing the risk for CAD and consecutive heart failure (30, 31). As another limiting factor, the study employed a retrospective approach, providing limited insight into the clinical further development of MACE or other risk factors beyond those assessed earlier by a dedicated questionnaire. Additionally, the use of various cardiac CT acquisition techniques could influence the radiomics results, so this should be addressed in a prospective setting. In the end, the leakage-free AUC of 0.59–0.65 indicated only a moderate rather than strong discrimination. Given this limitation in combination with the single-center nature, the limited sample size, lack of external validation and potential confounding, further prospective multi-center studies examining a multifactorial approach to clinical risk factors should be conducted to better understand the factors influencing textural changes in EAT, ultimately leading to a higher risk of MACE. The findings should be considered exploratory and hypothesis-generating.

## 5 Conclusion

In conclusion, PCCT-derived radiomic analysis of epicardial adipose tissue was associated with arterial hypertension in patients without coronary calcification or stenosis. These findings extend previous observations from EICT (7, 8), supporting the concept that cardiovascular risk factors are reflected in quantitative adipose tissue characteristics. As EAT is associated with MACE rate (1, 2) radiomic characterization of EAT may provide additional insight into early tissue remodeling related to cardiovascular risk. Larger multicenter studies with external validation and longitudinal outcome assessment are needed to determine the clinical relevance of EAT radiomic biomarkers and their relationship to future major adverse cardiovascular events.

In the future, PCCT-derived EAT radiomics may be used as a personalized risk stratification for the development of cardiovascular events, by providing quantitative imaging biomarkers of early cardiovascular disease.

## Data Availability

The raw data supporting the conclusions of this article will be made available by the authors, without undue reservation.
